# The naphthalene catabolic protein NahG plays a key role in hexavalent chromium reduction in *Pseudomonas brassicacearum* LZ-4

**DOI:** 10.1038/s41598-017-10469-w

**Published:** 2017-08-29

**Authors:** Haiying Huang, Xuanyu Tao, Yiming Jiang, Aman Khan, Qi Wu, Xuan Yu, Dan Wu, Yong Chen, Zhenmin Ling, Pu Liu, Xiangkai Li

**Affiliations:** 1 MOE Key Laboratory of Cell Activities and Stress Adaptations, School of Life Sciences, Lanzhou University, Lanzhou, Gansu 730000 P.R. China; 2 School of Life Sciences, Lanzhou University, Lanzhou, 730000 P.R. China

## Abstract

Soil contamination by PAH and heavy metals is a growing problem. Here, we showed that a new isolate, *Pseudomonas brassicacearum* strain LZ-4, can simultaneously degrade 98% of 6 mM naphthalene and reduce 92.4% of 500 μM hexavalent chromium [Cr (VI)] within 68 h. A draft genome sequence of strain LZ-4 (6,219,082 bp) revealed all the genes in the naphthalene catabolic pathway and some known Cr (VI) reductases. Interestingly, genes encoding naphthalene pathway components were upregulated in the presence of Cr (VI), and Cr (VI) reduction was elevated in the presence of naphthalene. We cloned and expressed these naphthalene catabolic genes and tested for Cr (VI) reduction, and found that NahG reduced 79% of 100 μM Cr (VI) in 5 minutes. Additionally, an *nahG* deletion mutant lost 52% of its Cr (VI) reduction ability compared to that of the wild-type strain. As *nahG* encodes a salicylate hydroxylase with flavin adenine dinucleotide (FAD) as a cofactor for electron transfer, Cr (VI) could obtain electrons from NADH through NahG-associated FAD. To the best of our knowledge, this is the first report of a protein involved in a PAH-degradation pathway that can reduce heavy metals, which provides new insights into heavy metal-PAH contamination remediation.

## Introduction

Environmental pollution caused by heavy metals and polycyclic aromatic hydrocarbons (PAHs) is an enormous environmental problem^1^. Due to their wide use in various industries, such as tanning, plating, pigment manufacturing, oil refining, and nuclear weapon production, the PAH naphthalene and hexavalent chromium [Cr (VI)] are common environmental pollutants^2, 3^. Naphthalene, which is the simplest polycyclic aromatic compound, has been classified as a priority toxic pollutant by the US Environmental Protection Agency^4^. Chromium is an important industrial material, and hexavalent chromium is a serious environmental threat due its toxic effects on humans and biodiversity^5^. These chemical compounds are deposited in soil and sediment, and when they reach high concentrations, they become harmful to the environment and human health^6^.

Decontamination of PAHs and heavy metals is a complex problem, as conventional remediation methods, such as chemical degradation of these organic pollutants and precipitation of chromium by transforming Cr (VI) into poorly soluble trivalent chromium [Cr(III)], may cause further environmental damage^7, 8^. Microbial remediation is a tempting solution for polluted environments that has been extensively studied. Bacteria that degrade toxic naphthalene into H_2_O and CO_2_ have been shown to be very useful for ameliorating naphthalene contamination. In one study, a bacterial consortium collected by enrichment culture was able to degrade 1,000 mg/L of naphthalene within 24 h^9^. In addition, a number of bacteria have the ability to reduce Cr (VI). For example, *Bacillus* sp. RE was shown to reduce more than 95% of 40 μg/mL Cr (VI) within 72 h^10^, and *Bacillus* sp., as well as *Leucobacter* sp. and *Exiguobacterium* sp. have potential for Cr (VI) remediation because they can tolerate and reduce high concentrations of Cr (VI)^11^. Rapid microbial reduction of Cr (VI) to Cr (III) creates a chemical with reduced mobility and toxicity. Although there are some microbes that can reduce Cr (VI), and others that can degrade PAHs, bioremediation of sites polluted with Cr (VI) and PAHs is likely to be limited to a single strain, because Cr (VI) is toxic to naphthalene-degrading bacteria and vice versa^12^. In addition, previous studies have demonstrated that Cr (VI) inhibits the biodegradation of organic pollutants^12–14^.

Numerous studies have been conducted to elucidate the enzymes involved in chromium reduction and naphthalene degradation and the genes encoding them in both gram-negative and gram-positive bacteria that can remediate naphthalene and chromium^15–17^. Chromate reductases are found in many bacterial strains, such as YieF and NfsA in *Escherichia coli* ^18, 19^, ChrR in *Pseudomonas putita* ^20^, and NfrA in *Bacillus subtilis* ^21^. The genes in the naphthalene-degrading pathway have been identified, including the upper pathway operon, which encodes the enzymes involved in the conversion of naphthalene to salicylate (*nahAaAbAcAdBFCED*), and the lower pathway operon, which encodes the enzymes involved in the conversion of salicylate to tri-carboxylic acid cycle intermediates by the meta cleavage pathway enzymes (*nahGTHINLOMKJ*)^22^. In our previous study, we have proved *Pseudomonas gessardii* LZ-E could degrade naphthalene and reduce Cr (VI) simultaneously. When strain LZ-E degrades naphthalene, the intermediate catechol is able to reduce Cr (VI) abioticly^23^. However, no studies have demonstrated the naphthalene degradation enzymes can reduce Cr (VI) directly.

The area along the Yellow River near the PetroChina Company is severely polluted by waste water containing various substances, including PAHs and heavy metals. Using a systematic approach aimed at remediating the effects of these co-pollutants, we isolated bacteria from this site. Among the isolates, one strain, LZ-4, simultaneously catabolized PAHs and reduced hexavalent chromium. No previous study has shown that these two process are related. In this study, we also elucidated the mechanism through which the naphthalene catabolic protein NahG elevated Cr (VI) reduction in strain LZ-4 by determining the chemical and enzymatic reactions.

## Results

### Phylogenetic analysis and phenotypic characterization of strain LZ-4

To isolate naphthalene-degrading bacterial strains, the final enrichment culture was diluted in BH medium and plated on BH agar plates sprayed with naphthalene. From the final dilution, 18 strains with different morphotypes were isolated (data not shown). Among these 18 isolates, strain LZ-4 showed the most efficient naphthalene degradation and Cr (VI) reduction (data not shown). Thus, this strain was chosen for further studies. Gram staining, 16 S rRNA gene sequencing, and Vitek revealed that strain LZ-4 was a rod-shaped, gram-negative bacterium with 98.27% sequence similarity to *Pseudomonas brassicacearum*. A phylogenetic tree was generated based on 16 S rRNA gene sequences by the neighbour-joining method (Supplementary Figure S1A). The 16 S rRNA gene sequence was deposited in GenBank (accession number, KM 453978). Whole genome analysis also showed that strain LZ-4 was closely related to *P. brassicacearum*, As the 16 S rRNA and whole genome sequence analyses showed that strain LZ-4 was very closely related to P. brassicacearum, we have designated this strain *P. brassicacearum* LZ-4 (Supplementary Figure S1B and C).

### Cr (VI) reduction by strain LZ-4

**Figure 1:**
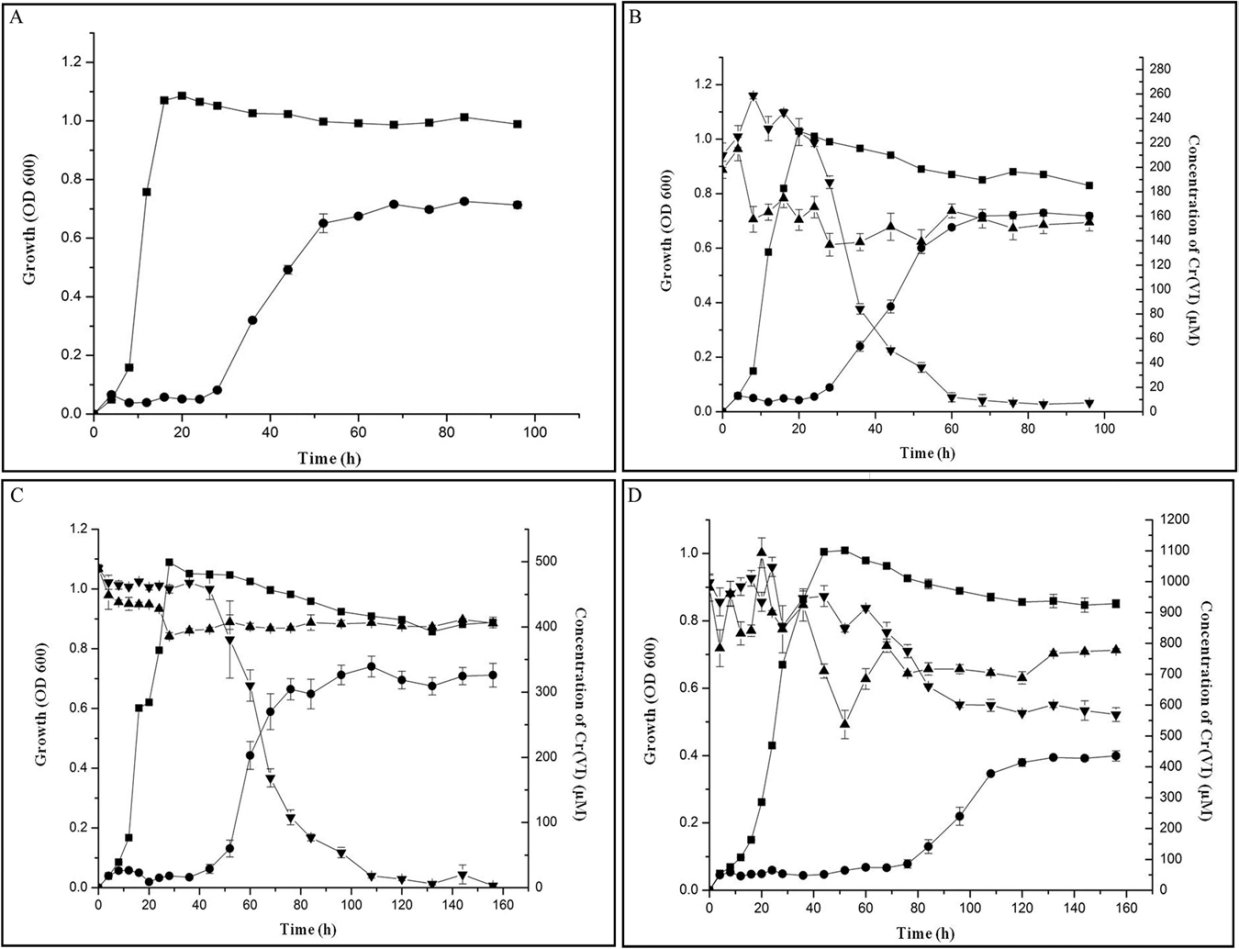
Growth of strain LZ-4 in BH medium containing different concentrations of Cr (VI), (**A**) 0 μM, (**B**) 200 μM, (**C**) 500 μM, and (**D**) 1 mM and either naphthalene (●) or glucose (■) as the sole carbon source. Cr (VI) reduction by strain LZ-4 in BH medium containing 200 μM (**B**), 500 μM (**C**), or 1 mM (**D**) Cr (VI) in the presence of naphthalene (▼) or glucose (▲) as the sole energy source. Strain LZ-4 in BH medium alone, with no added energy source, was used as the negative control in (**A**). Data are the mean of three separate experiments, and the error bars are the standard deviation. The left vertical, right vertical, and horizontal axes are the OD_600_, remaining Cr (VI), and time (h), respectively. Figure 1b,c, and d show that Cr (VI) was indeed reduced.

The minimum inhibitory concentration of Cr (VI) for strain LZ-4 when grown in BH medium using naphthalene as the sole carbon source was 1 mM. The OD_600_ of strain LZ-4 reached 0.72 when naphthalene was added as the carbon source and 1.1 when glucose was added as the carbon source (Fig. 1A), suggesting that strain LZ-4 grows better when utilizing glucose as a carbon source. To investigate Cr (VI) reduction in strain LZ-4, the strain was cultured with different concentrations of Cr (VI) for 68 h. In 200 μM Cr (VI), strain LZ-4 reduced 96.2% of the Cr (VI) present when using naphthalene as the sole carbon source, whereas 25% of the Cr (VI) was reduced when glucose was used as the sole carbon source (Fig. 1B). Similarly, strain LZ-4 reduced 500 μM Cr (VI) by 92.4% in the presence of naphthalene, but by only 21% in the presence of glucose (Fig. 1C). When the concentration of Cr (VI) was increased to 1,000 μM, growth of strain LZ-4 was obviously repressed due to chromate toxicity, and only 42% of the Cr (VI) was reduced. Interestingly, 1,000 μM Cr (VI) was still only reduced by 22% in the presence of glucose (Fig. 1D). Collectively, these observations provide a direct demonstration that strain LZ-4 can efficiently reduce Cr (VI) while using naphthalene as the sole carbon source.

### Genome sequencing and determining the naphthalene catabolic pathway

**Figure 2:**
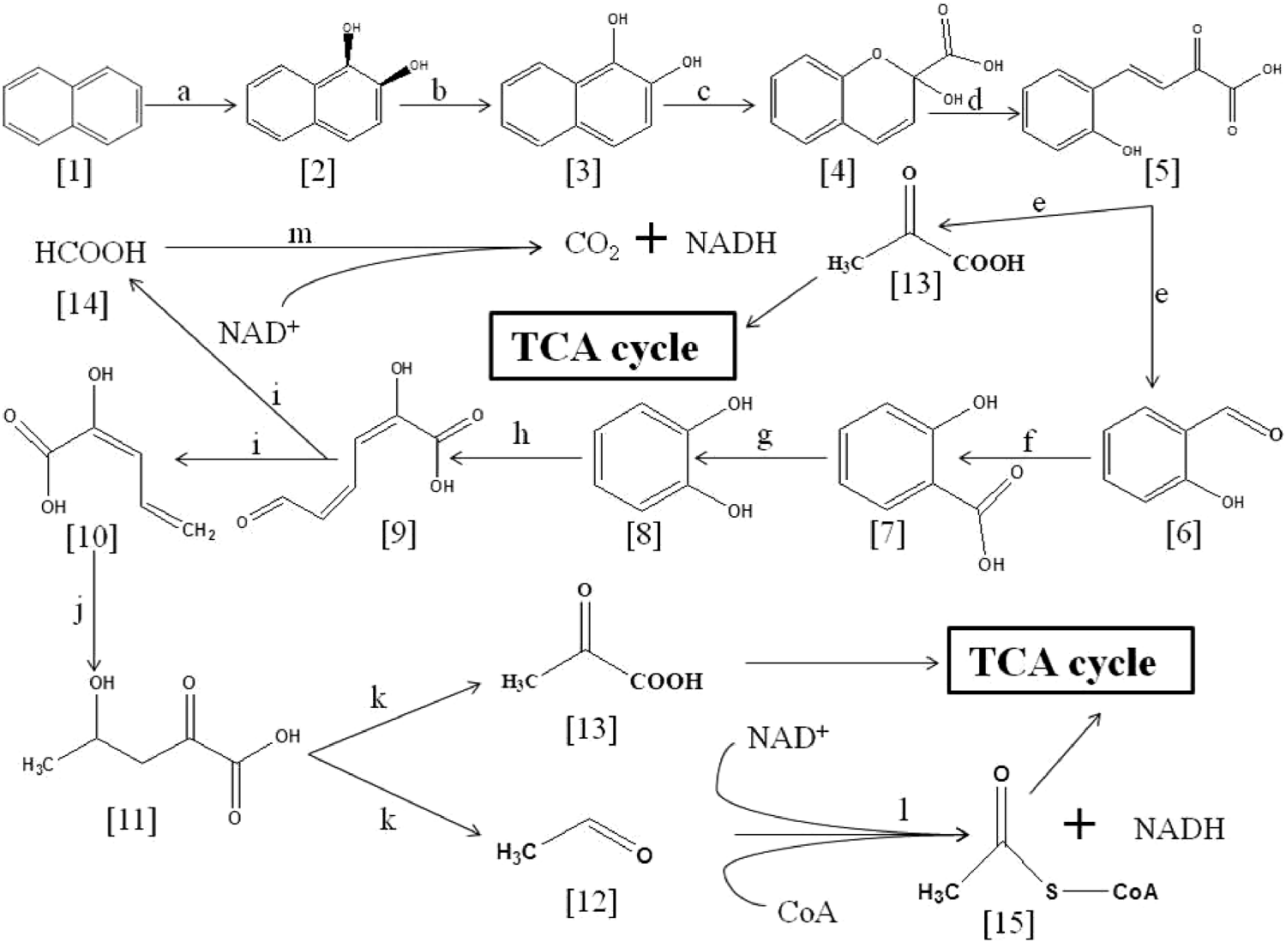
Predicted naphthalene degradation pathway in strain LZ-4. Chemical designations: [1], naphthalene^60^, cis-1,2-dihydronaphthalene-1,2-diol; [3], naphthalene-1,2-diol[4],2-hydroxychromene-2-carboxylate; [5], trans-o-hydroxybenzylidenepyruvate; [6], salicylaldehyde; [7], salicylate; [8], catechol; [9], 2-hydroxymuconate semialdehyde; [10], 2-hydroxy-2,4-pentadienoate; [11], 4-hydroxy-2-oxopentanoate; [12], acetaldehyde; [13], pyruvate; [14], formate; and [15], acetyl-CoA. Enzyme designations: (a) naphthalene 1,2-dioxygenase (ORFs 05125, 05126, 05127, and 05128, NahA); (b) cis-1,2-dihydro-1,2-dihydroxynaphthalene (ORF 05129, NahB); (c) 1,2-dihydroxynaphthalene dioxygenase (ORF 05131, NahC); (d) 2-hydroxychromene-2-carboxylate isomerase (ORF 03217, NahD); (e) trans-o-hydroxybenzylidene pyruvate hydratase-aldolase (ORF 05132, NahE); (f) salicylaldehyde dehydrogenase (ORF 05130, NahF); (g) salicylate hydroxylase (ORF 05164, NahG); (h) catechol 2,3-dioxygenase (ORF 05161, NahH); (i) 2-hydroxymuconate-semialdehyde hydrolase (ORF 05159, NahI); (j) 2-keto-4-pentenoate hydratase (ORF 05158, NahJ); (k) 4-hydroxy 2-oxovalerate aldolase (ORF 05156, NahK); (l) acetaldehyde dehydrogenase (ORF 05157, NahL); and m, formate dehydrogenase (ORFs 00216, 00217, and 00218, NahM^61–63^.

To understand the mechanism of simultaneous naphthalene degradation and Cr (VI) reduction, the genome of strain LZ-4 was sequenced by a whole genome shotgun approach. The total size of all contigs was 6,219,082 base pairs, with an average G + C content of 60.08%, and 5,464 open reading frames (ORFs; average length, 979 bp). Of the 5,464 ORFs, 3,882 were annotated based on matches in the GO database (Supplementary Table S2). GO cluster analysis showed that the 3882 proteins were distributed in the categories of biological process, cellular component, and molecular function (Supplementary Figure S2A), and 4,638 protein coding genes were distributed among 22 COG functional categories (Supplementary Figure S2B). The genome of strain LZ-4 was compared to the genomes of other related *Pseudomonas* strains, including *Pseudomonas putida* F1, *Pseudomonas fluorescens* Pf0-1, *Pseudomonas* sp. UW4, and *Pseudomonas brassicacearum* NFM421. The genome sequence was submitted to GenBank, and the whole genome shotgun project has been deposited at DDBJ/EMBL/GenBank (accession number, JNCR 00000000). The genome sequencing data revealed that strain LZ-4 has a complete set of naphthalene degradation pathway genes (Fig. 2, Table 1).

**Table 1:** Genes in the naphthalene degradation pathway in *Pseudomonas brassicacearum* LZ-4.

Gene	Putative function	Nucleotide identity
*nahAa*	Naphthalene 1,2-dioxygenase ferredoxin	100%
*nahAb*	Naphthalene 1,2-dioxygenase subunit	100%
*nahAc*	Naphthalene 1,2-dioxygenase subunit alpha	100%
*nahAd*	Naphthalene1,2-dioxygenase iron sulfur protein component small subunit	100%
*nahB*	1,2-dihydroxy-1,2-dihydronaphthalene dehydrogenase	99%
*nahC*	1,2-dihydroxynaphthalene dioxygenase	100%
*nahD*	2-hydroxychromene-2-carboxylate isomerase	100%
*nahE*	Trans-O-hydroxybenzylidenepyruvate hydratase-aldolase	100%
*nahF*	Salicylaldehyde dehydrogenase	99%
*nahG*	Salicylate hydroxylase	100%
*nahH*	Metapyrocatechase	100%
*nahI*	2-hydroxymuconicsemialdehyde dehydrogenase	100%
*nahL*	2-oxypent-4-enoate hydratase	100%
*nahJ*	4-oxalocrotonate tautomerase	100%
*nahK*	2-oxo-3-hexenedioate decarboxylase	100%
*nahM*	4-hydroxy 2-oxovalerate aldolase	100%
*nahN*	2-hydroxymuconic semialdehyde hydrolase	100%
*nahO*	acetaldehyde dehydrogenase	100%
nahR	nahR	100%

Nucleotide identity was determine by BLAST.

### Crude enzyme activity assay

**Figure 3:**
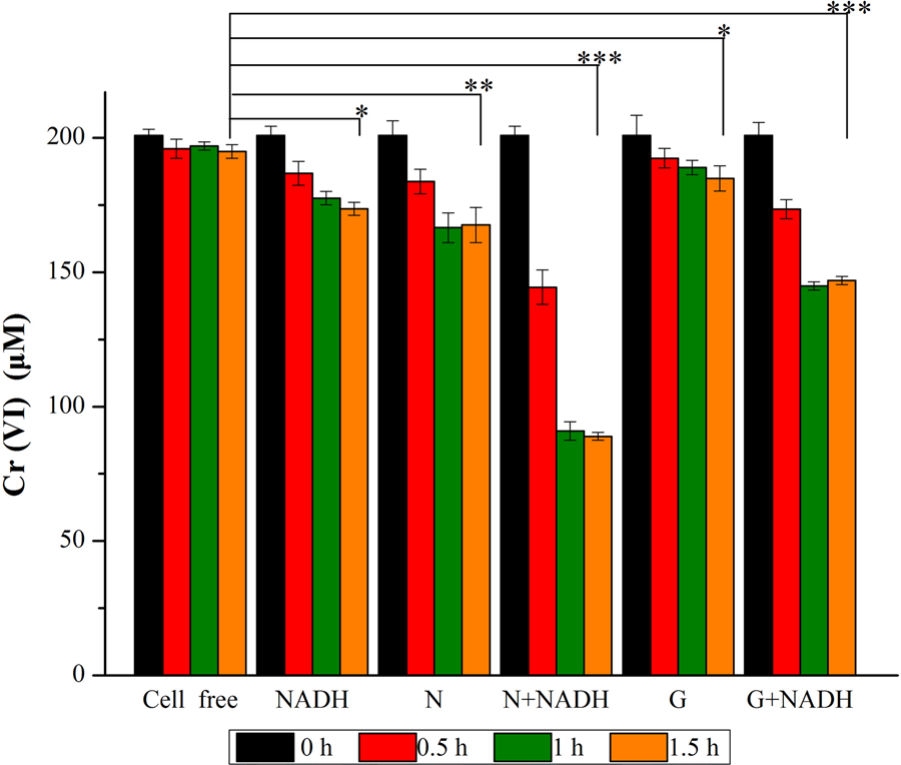
Crude enzyme activity assay showing that the enzymes induced in the presence of naphthalene (N) and glucose (G) can reduce Cr (VI) using NADH as an electron donor. Cell free, NADH, N, and G are the negative controls. Significant differences of Cr (VI) residue between each treatment group (N, G, N + NADH, and G + NADH) and the control group (cell-free) at 1.5 h were indicated by *p < 0.05,**p < 0.01, and ***p < 0.001.

Next, we tested whether the identified enzymes involved in the naphthalene catabolic pathway also contributed to Cr (VI) reduction during naphthalene degradation in strain LZ-4. First, the Cr (VI)-reducing ability of a crude enzyme preparation from a culture grown in medium containing naphthalene or glucose was assayed. Cells were harvested and broken by ultrasonic treatment after 0.5, 1, and 1.5 h of incubation with 200 μM Cr (VI) and NADH. After 1.5 h of incubation, the Cr (VI) was reduced by 56% (to 88 μM) in the presence of naphthalene, whereas the Cr (VI) was reduced by 26.5% (to 147 μM) in the presence of glucose (Fig. 3). These results showed that the enzymes induced in the presence of naphthalene reduced Cr (VI) more efficiently than those induced in the presence of glucose.

### Cr (VI) reduction by NahG

**Figure 4:**
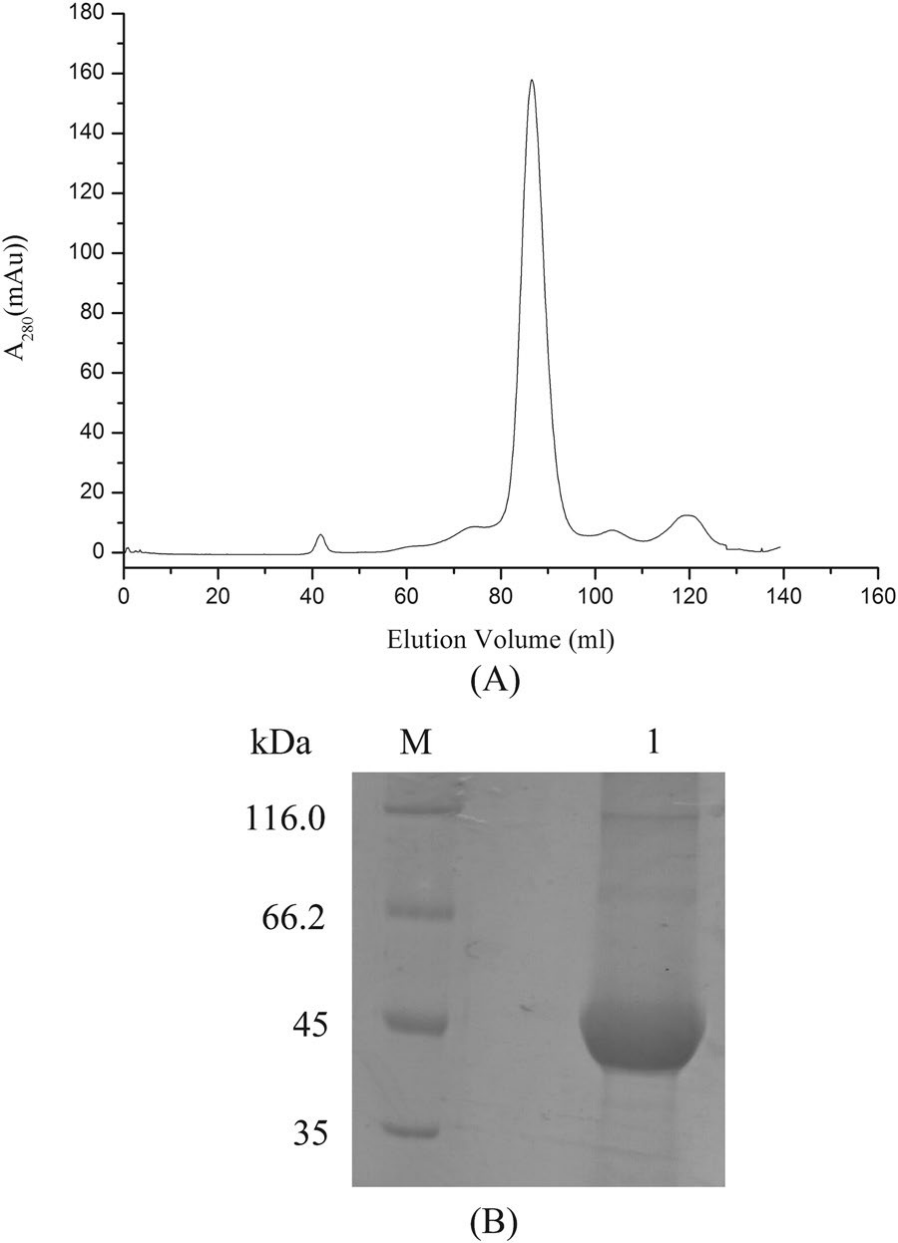
NahG protein purification by Ni2+ -nitrilotriacetate affinity and gel filtration chromatography (**A**). The purity of the NahG preparation (Lane 1) was checked by 10% SDS-PAGE (**B**).

**Figure 5:**
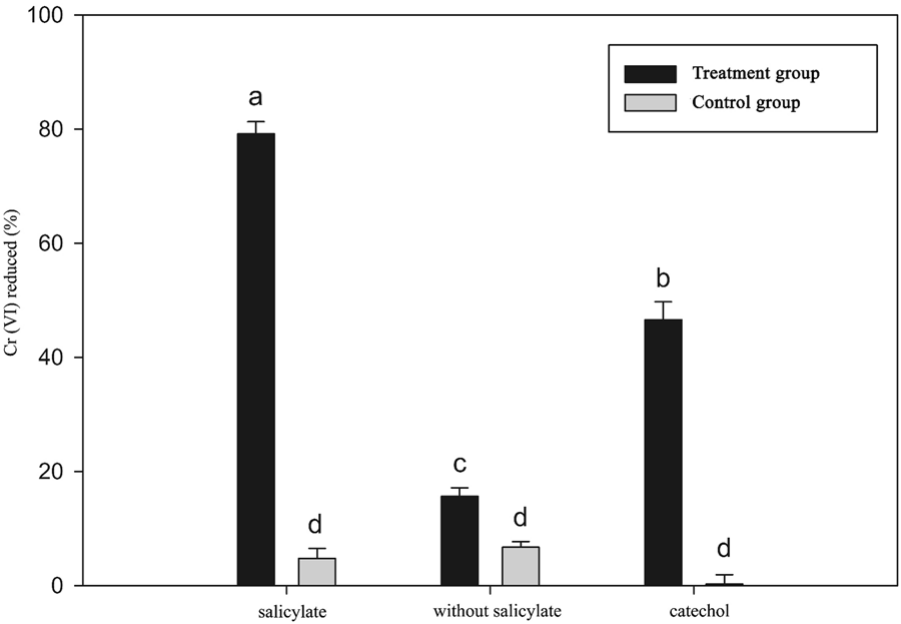
Cr (VI) reduction rates with or without salicylate and with or without catechol. Cr (VI) reduction experiments were conducted. Reactions in the treatment group contained 10 µM NahG, and reactions in the control group did not. The salicylate group reactions also contained 300 µM salicylate, 10 µM FAD, 100 µM Cr (VI), 200 µM NADH, and 20 mM HEPES (pH 7.0). The without salicylate group contained all components except salicylate. The catechol group contained 300 µM catechol, 10 µM FAD, 100 µM Cr (VI), 200 µM NADH, and 20 mM HEPES (pH 7.0). The Cr (VI) concentration was determined after incubation at 30 °C for 1 min. The percentage of reduced Cr (VI) was calculated. Each group included five replicates. a, b, c, and d on the top of the bar indicate that the mean is significantly different at 95% level of confidence.

All the naphthalene catabolic genes identified in the strain LZ-4 genome were upregulated by naphthalene (data not shown). Among these genes, *nahG* encodes an oxidoreductase that converts salicylate to catechol using a FAD as cofactor. In addition, NahG can also transfer electrons from NADH to oxygen. We hypothesized that this transfer of electrons from NADH could promote Cr (VI) reduction. Based on the protein sequence predicted from the nucleotide sequence, *nahG* was cloned and expressed in *E. coli*, and the protein was purified by Ni^2+^-nitrilotriacetate affinity and gel filtration chromatography. The purity of the NahG protein preparation was assessed by 10% SDS-PAGE, and was determined to be >95% (Fig. 4). The main function of NahG, the conversion of salicylate to catechol, was confirmed by HPLC (Supplementary Figure S3). We then investigated the ability of NahG to reduce Cr (VI). The reaction mixture contained 10 μM NahG, FAD, 100 μM Cr (VI), and 200 μM NADH in 20 mM HEPES (pH 7.0). The final concentration of Cr (VI) revealed that NahG can reduce Cr (VI) aerobically in the presence of NADH and salicylate or catechol. In the presence of NahG and salicylate, 79% of the Cr (VI) was reduced, whereas only 17% of the Cr (VI) was reduced in reactions containing NADH but not salicylate or catechol. In the presence of catechol and NahG, 46% of the Cr (VI) was reduced (Fig. 5). To confirm the effect of NahG, reactions were incubated without NahG, and little no Cr (VI) reduction was observed (Fig. 5). According to the above results, the NahG protein can efficiently reduce Cr (VI) in the presence of salicylate. There are two possible mechanisms for chromate reduction by NahG. First, NahG reduces chromate by directly transferring electrons from NADH to chromate. Second, NahG indirectly reduces chromate through the conversion of salicylate to catechol, which can reduce chromate. Both the direct and indirect mechanisms lead to efficient chromate reduction. The present data suggested that the NahG protein uses NADH as electron donor and could remediate naphthalene and Cr (VI) simultaneously.

### Reduction of chromate by the *P. fluorescens* LZ-4 *ΔnahG* strain and complemented nahG + *ΔnahG* strain

**Figure 6:**
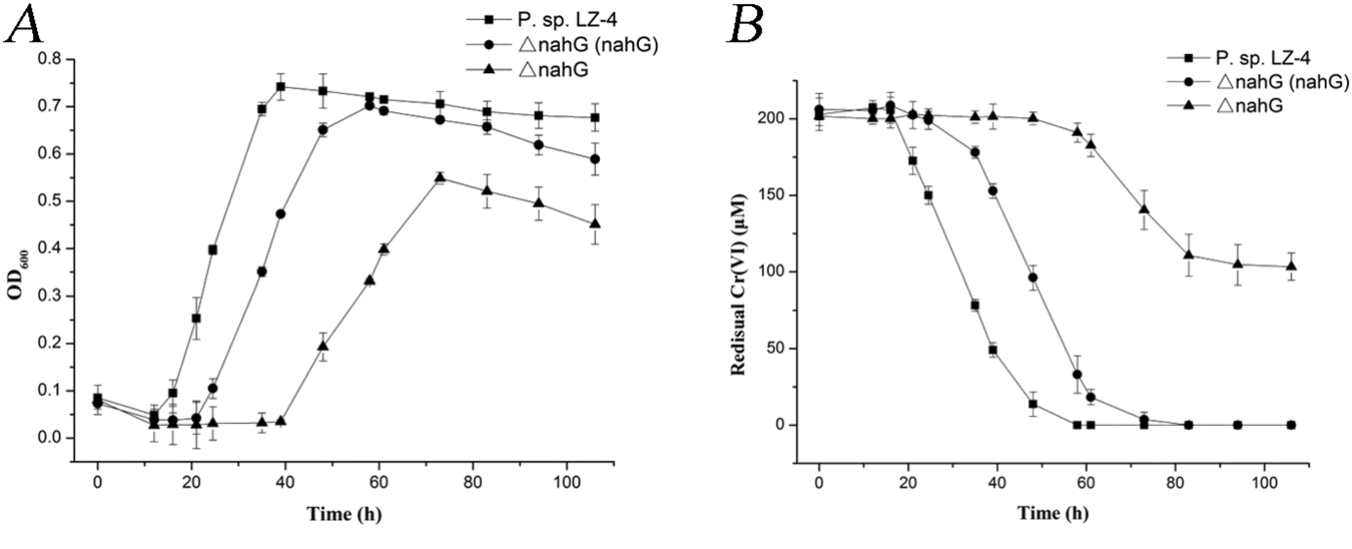
Growth curve and Cr (VI) reduction by the *Pseudomonas brassicacearum LZ-4* wild-type strain (*P*.sp. LZ-4), *nahG* deletion mutant (ΔnahG) strain, and *nahG*-complemented *nahG* deletion mutant strain [ΔnahG (nahG)]. (**A**) Growth curve of *P*.sp. LZ-4, ΔnahG, and ΔnahG (nahG). (**B**) Chromate reduction by *P*.sp. LZ-4, ΔnahG, and ΔnahG (nahG) in BH medium containing 200 μM Cr (VI).

To investigate the hypothesis that NahG is involved in both the naphthalene degradation pathway and Cr (VI) reduction in strain LZ-4, an *nah*G deletion mutant was constructed. Compared to the wild-type LZ-4 strain, the growth of the *nah*G deletion mutant strain in BH medium containing naphthalene and 200 μM Cr (VI) was greatly reduced, and the strain showed a log phase delay. In addition, the maximum OD_600_ in stable phase was only ~0.48 (Fig. 6A). This may be because the *nahG* gene encodes a key enzyme in naphthalene degradation.

The wild-type strain reduced almost all of the added Cr (VI) within 60 h, whereas the mutant strain only reduced 48.75% of the added Cr (VI) within 100 h (Fig. 6B), showing a partial loss of Cr (VI) reduction ability. The complemented ΔnahG strain (*nahG*+) was similar to the wild-type strain and efficiently reduced 98.54% of the Cr (VI) within 70 h, indicating that the *nahG* deletion was the cause of the observed Cr (VI) reduction defect.

## Discussion

Remediation of sites with combined pollution is a growing environmental challenge. Bioremediation of PAHs and hexavalent chromium has not yet been achieved, in part because Cr (VI) is toxic to many naphthalene-degrading bacteria and inhibits the biodegradation of organic pollutants^12–14^. In addition, PAHs can suppress or reduce heavy metal reduction in most bacteria^24, 25^. However, a previous study showed that some PAH-degrading bacteria can survive in the presence of other pollutants^26^, and that *Pseudomonas gessardii* LZ-E has been reported as a potential candidate for the remediation of combined pollution, because it can simultaneously degrade naphthalene and reduce Cr (VI) without inhibition^23^. In addition, one study showed that a co-culture of *Bacillus* sp. and *Sphingomonas paucimobilis* can simultaneously degrade naphthalene and reduce Cr (V1)^27^. However, to date, no single study demonstrated that naphthalene degradation can promote Cr (VI) reduction in *Pseudomonas* sp. In the present study, we showed that a new isolate, strain LZ-4, simultaneously catabolized the PAH naphthalene and reduced hexavalent chromium. In addition, strain LZ-4 showed a high level of tolerance to PAHs and heavy metals and reduced much more chromate during naphthalene degradation (in the presence of naphthalene; Fig. 1)’. The draft genome sequence of strain LZ-4 was compared with other *Pseudomonas* species through reciprocal BLAST searches^28^. When compared to *P. brassicacearum* strain NFM 421, *Pseudomonas* sp. UW4, *P. fluorescens* Pfo-1, and *P. putida* F1, 2,767 (50.99%), 2,763 (50.91%), 2,713 (50.0%), and 2,608 (48.06%) of the predicted ORFs in *Pseudomonas* sp. strain LZ-4 had homologs in these bacteria; therefore, strain LZ4 contained 2,157 unique predicted ORFs (>39.75%). (Supplementary Figure S1B and C). The NahABFCDEF proteins in strain LZ-4 convert naphthalene into salicylate, which is directly converted to catechol by the action of a single monooxygenase (salicylate hydroxylase, encoded by ORF 05164, *nahG*; Fig. 2). Similar pathways have been reported in *P. putida* G7^29^ *P. putida* ^30^, *P. stutzeri* ^31^, and strain CA10^32^. The genes that are regulated by naphthalene probably contribute to the degradation of naphthalene^33^. Naphthalene-degrading microorganisms have attributes because of the occurrence of redundant relative genes, such as dehydrogenase, dioxygenase and monooxygenase^34^. However, it is also possible that the Cr (VI) reduction through the naphthalene catabolic pathway in strain LZ-4 is a new phenomenon.

Our chemical reaction assays showed that catechol is a key intermediate for both naphthalene degradation and Cr (VI) remediation, as catechol is an intermediate chemical product of the naphthalene degradation pathway and functions as the primary reductant for Cr (VI) reduction, even at low concentrations. Catechol generated during naphthalene degradation was previously shown to reduce Cr (VI) to Cr (III) in *Pseudomonas gessardii* LZ-E^23^, and the same phenomenon was observed in strain LZ-4. In addition, we found that strain LZ-4 can still reduce Cr (VI) without intermediates, and the naphthalene degradation enzymes in strain LZ-4 stimulate Cr (VI) reduction (Fig. 3). A crude extract of the enzymes induced in the presence of naphthalene can reduce up to 52% of Cr (VI) using NADH as an electron donor (Fig. 3). NADH is a cofactor for Cr (VI) reduction in *Pseudomonas* sp.^32, 35, 36^, as it acts as electron donor for the conversion of Cr (VI) into less soluble Cr(III)^10^. Although, combined pollutants (heavy metals and PAHs) also affect microbial enzymatic activity in ecological systems^37^, the results of the current study suggested that strain LZ-4 can interact with more than one contaminant, which is of particular interest for bioremediation of combined pollutants.

The naphthalene-degrading protein NahG participates in Cr (VI) reduction in strain LZ-4, and as a purified protein, it reduced 79% of Cr (VI) within 1 min (Fig. 5). Several naphthalene carbolic proteins have been identified in *Pseudomonas* sp., including NahR, NahG, NahU, and NahW^15, 38–40^, and these proteins imparted resistance to the toxic effects of naphthalene, hence these bacterial cells can persistent in soils where naphthalene occurs at high concentrations^41^.

Cr (VI) reduction had not been shown to be associated with the naphthalene-degradation pathway. Here, we confirmed that NahG converts salicylate to catechol (Supplementary Figure S3), and we showed that Cr (VI) reduction was associated with the naphthalene catabolic pathway. To verify the role of NahG, a *ΔnahG* mutant was constructed, and its growth and Cr (VI) reduction ability were compared to those of a wild-type strain. Both growth and Cr (VI) reduction were reduced in the *nahG* mutant when compared to the wild-type strain. In contrast, the phenotype of the complimented *nahG* deletion mutant was similar to that of wild type in terms of both growth and Cr (VI) reduction.

According to a previous report^42^, almost all chromate reductases contain a cofactor, such as FAD or FMN. NahG also contains FAD as a cofactor; thus, it might also function as a chromate reductase. NahG and homologous enzymes are flavoproteins^43^, and chromate reductases are also flavoproteins^42^. There is also evidence to support the idea that many soluble flavoproteins with unrelated metabolic functions can catalyze chromate reduction^36^. However, chromate can form complexes with organics, and the reduction mechanism is not yet clear^44^. As previous studies showed, NahG can convert salicylate to catechol, generating carbon dioxide and water when using NADH as the electron donor, as show in the following reaction: Salicylate + NADH + 2 H^+^ + O_2_ → Catechol + NAD^+^ + H_2_O + CO_2_^45, 46^. In our study, chromate was also added into the medium. We hypothesized that during the conversion of salicylic acid to catechol catalysed by NahG, electrons from NADH are not only transferred to oxygen, but also to Cr^6+^. Thus, the reaction could be revised as follows: Salicylate + NADH + 2 H^+^ + O_2_/Cr^6+^ → Catechol + NAD^+^ + H_2_O/Cr^3+^ + CO_2_. The conversion of salicylate to catechol is important for chromate reduction by NahG, which can drive the transfer of electrons from NADH to Cr^3+^. This evidence confirmed that NahG, induced by naphthalene, plays a key role in Cr (VI) reduction. In addition, this study also provides new insights into microbial remediation of Cr (VI)/PAH combined contamination.

## Materials and Methods

### Soil sampling and media

Soil samples were collected at a 15-cm depth from one side of the Yellow River near the PetroChina Company in Lanzhou, China (36° 06′ N 103°39′ E). The pH and temperature of soil at the sample site were 5.5 and 18 °C, respectively, and samples were stored in sterile aluminium boxes at 4 °C^23^. Bushnell-Haas (BH) minimal medium with 1.5% NaCl was used for enrichment, isolation, and growth^47^. Naphthalene was purchased from Aladdin Chemistry Co., Ltd.

### Enrichment cultures and strain isolation

100 mg naphthalene, as the sole carbon and energy source, dissolved in cyclohexane to a final concentration of 1 mM, was added to an empty flask. The medium was not added to the flask until the solvent for naphthalene (cyclohexane) had evaporated^7, 48^. Then, 200 mL of BH minimal medium containing 1 mM Cr (VI) and a 2-g sediment sample were added to the flask. Similar cultures without naphthalene were used as a negative control. The bacterial cultures were incubated aerobically at 28 °C with shaking at 180 rpm. After incubation for 2 weeks, an aliquot (1 mL) of the initial enrichment culture was inoculated into a second enrichment culture at 1% [vol/vol], and incubated for another 2 weeks. Finally, 1% (vol/vol) of the medium from the second enrichment was inoculated into a third and final enrichment culture. To isolate naphthalene-degrading bacterial strains, the final enrichment was diluted in BH medium and plated on BH agar plates sprayed with naphthalene. Then, the colonies were cultured in BH medium containing 1 mM naphthalene.

### Identification and characterization of the isolated strain

For 16 S rRNA sequencing analysis, genomic DNA was extracted from the isolated strain with the mini BEST Bacterial Genomic DNA Extraction Kit (TAKARA BIOTECHNOLOGY, LTD.) according to the manufacturer’s instructions. The 16 S rRNA gene sequence was amplified using the universal bacterial primers 27 F (5′-AGAGTTTGATCCTGGCTCAG-3′) and 1492 R (5′-GGTTACCTTGTTACGACTT-3′) and cloned in the pMD18-T vector (TAKARA BIOTECHNOLOGY, LTD.) for sequencing. The obtained sequence was analysed against the NCBI and EzTaxon databases to identify the most closely related species. Then, a phylogenetic tree was constructed with MEGA (version 4.0)^28^.

### Cr (VI) reduction assay

For the Cr (VI) reduction assay, 6 mM naphthalene or glucose, as a carbon source, was added to a sterile empty flask. Then, 1.5 mL of medium containing strain LZ-4, 150 mL of BH minimal medium, and Cr (VI) (at 200 μM, 500 μM, or 1000 μM) were added, and the flask was incubated at 28 °C with shaking at 180 rpm. Bacterial growth was monitored as the OD_600_, and culture medium without Cr (VI) was used as a negative control. All experiments were performed in triplicate. The concentration of Cr (VI) was measured with the calorimetric reagent 1,5-diphenylcarbazide (DPC) according to a previously reported method^49^.

### Draft genome sequencing and annotation of strain LZ-4

The whole genome of strain LZ-4 was sequenced with an Illumina HiSeq. 2000 sequencer. DNA libraries were constructed using NextEra technology and sequenced using a 2 × 100 nucleotide paired-end strategy. All reads were pre-processed to remove low-quality artificial bases^50^. After filtering, the remaining reads were assembled with SOAPdenovo (http://soap.genomics.org.cn, version 1.05). ORF prediction was performed with Glimmer 3.0 (http://www.cbcb.umd.edu/software/glimmer/). For annotation, the predicted protein sequences of genes were aligned with sequences in the Nr, String, and GO databases by BLAST (BLAST 2.2.24+). For comparative genomics, the genome sequences of *Pseudomonas putida* F1 (NC_002947.4), *Pseudomonas fluorescens* Pf0-1 (NC_007492.2), *Pseudomonas* sp. UW4 (NC_019670.1), and *Pseudomonas brassicacearum* NFM421 (NC_015379.1) were downloaded from the NCBI database. Genome structure was compared with MUMmer software.

### Cr (VI) reduction assay with crude extract

Strain LZ-4 was cultivated in BH medium containing naphthalene or glucose as the sole carbon source for 48 h. Then, the cells were collected and washed, and crude Cr (VI) reduction activity was determined as described previously^51^.

### Construction of a *ΔnahG* strain and complemented (nahG+) Δ*nahG* strain

An *nahG* deletion mutation strain was constructed according to a previously described homologous recombination gene knockout method using the pK18mobsacB plasmid^52^. The plasmid used for *nahG* knockout was constructed as follows: 500-bp fragments upstream and downstream of *nahG* were amplified from strain LZ-4 genomic DNA by PCR. Then, these upstream and downstream fragments were linked using an overlapping PCR method to generate the *ΔnahG* fragment, and then the *ΔnahG* fragment and pK18mobsacB plasmid were digested with BamHI and HindIII and ligated to generate pKΔnahG. *Escherichia coli* S17 cells were transformed with pKΔnahG, which contains the kanamycin resistance gene for positive selection. Then, pKΔnahG was transferred from *E. coli* S17 to strain LZ-4 by conjugation, according to a previously described procedure^52^. Strain LZ-4 pKΔnahG conjugates were first selected on kanamycin. Then, *ΔnahG* recombinants were selected on sucrose (due to a loss of the sucrose sensitivity conferred by *sacB* in the plasmid The bacterial strains and plasmids used in this study are shown in Supplementary Table S1.

### Construction, expression, and purification of NahG protein

The *nahG* gene was amplified from the *P. brassicacearum* strain LZ-4 genome by PCR, using the forward primer (5′-CATGCC **ATG** GGC ATGAAAAACAATAAACCTGGCTTGC-3′) and reverse primer (5′-CCGCTCGAG TCACCCTTGACGTAGCACACC-3′), which introduce **NcoI** and **XhoI** restriction sites (underlined), respectively. The start codon is shown in bold. The amplified fragment was cloned into a pET28b-derived vector with a C-terminal 6 × His-tag for expression in *Escherichia coli*. The recombinant plasmid was then transformed into *E. coli* BL21 (DE3) cells (Novagen) and grown in Luria Bertani medium (LB) to an OD_600_ of 0.8. The cells were harvested by centrifugation and resuspended in 20 mM Tris-HCl, pH 8.0 containing 100 mM NaCl. After 30 min of sonication and centrifugation at 12,000 × *g*, the supernatant was collected and loaded onto Ni^2+^-nitrilotriacetic acid affinity resin (Ni-NTA; Qiagen) equilibrated with buffer (20 mM Tris-HCl pH 8.0, and 100 mM NaCl). The target protein was eluted with 300 mM imidazole in the same buffer and further purified with a gel filtration chromatography column (HiLoadTM 16/60 Superdex TM 200; GE Healthcare) equilibrated with 20 mM Tris-HCl pH 8.0 and 100 mM NaCl. The peak fractions were pooled and concentrated to a final concentration of 50 mg/mL for further use^53^.

### Salicylate metabolism and chromate reduction by NahG

High performance liquid chromatography (HPLC; Agilent 1260 Infinity) was used to verify that NahG is involved in the conversion of salicylate to catechol^54^. The metabolites were separated with an Eclipse Plus C18 (4.6 mm × 250 mm). The mobile phase consisted of 30% methanol and 70% water, at a flow rate of 0.8 mL/min, and the fluorescence detector was set at 280 nm for the detection of naphthalene. The retention time of salicylic acid, catechol, FAD, and NADH were 1.767 ± 0.1 min, 4.196 ± 0.1 min, 1.079 ± 0.1 min, and 1.208 ± 0.1 min, respectively. A Cr (VI) reduction assay was conducted using NahG protein under aerobic conditions to determine whether the protein has the ability to reduce Cr (VI). The reaction mixtures contained 20 μM NahG protein, 20 μM FAD, 150 μM NADH, 100 μM Cr (VI), and 20 mM HEPES buffer (pH 7.0) in a volume of 2 mL. The reaction was started by adding the reactants, and Cr (VI) was measured at the indicated time points^55^.

### Quantitative PCR (q-PCR) analysis of *nahG*

Strain LZ-4 was grown aerobically in BH medium containing 6 mM naphthalene or glucose as the sole carbon source. The cells were harvested at an OD_600_ of 0.6. Then, the 10-mL cultures were centrifuged, and cell pellets were washed twice with sterile ddH_2_O. Total RNA was isolated with the SV total RNA isolation system (Promega) according to the manufacturer’s instructions. The isolated RNA was reverse transcribed with the PrimeScriptTM164 RT reagent Kit (TaKaRa, Dalian, China). Then, qPCR was carried out to determine *nahG* expression levels, and the 16 S rRNA gene was included as a control. The qPCR reaction system contained 5 μL of SYBR Green PCR Master Mix, 0.8 μL of primer (10 mM), 0.8 μL of cDNA (100 ng·μL^−1^), and sterile ddH_2_O to a final volume of 20 μL. The primers used are listed in Table 2. The qPCR cycle conditions were as follows: an initial denaturation step at 95 °C for 10 min, followed by 40 cycles of denaturation at 95 °C for 15 sec, annealing at 58 °C for 30 sec, and elongation at 72 °C for 30 sec.

**Table 2:** Primers used in this study.

Primer	Sequence (5′ → 3′)
nahGqF1	CGGGATCCCGATGAAAAACAATAAACCTGGCTTGCGC
nahGqR1	GCATCGAGCAGCTGACTTGATTCCGTCGGC
nahGqF2	TCAAGTCAGCTGCTCGATGCCTTCGCGGG
nahGqR2	CCCAAGCTTGGGTCACCCTTGACGTAGCACACC
nahGF	TGACGGCCATATCCTCACTTT
nahFR	GTTCGGCTTCGGCTCACTA

### Statistical analyses

General statistical analyses were performed using parametric tests. Differences were considered statistically significant when P < 0.01, P < 0.05, and P < 0.001. For the crude enzyme activity experiments, significant differences of Cr (VI) residue between each treatment group (N (naphthalene), G (glucose), N + NADH, and G + NADH) and the control group (cell-free) at 1.5 h were determined by Student’s t-test. For levels of NahG protein in the Cr (VI) reduction experiment, the significance of the differences were determined by Tukey’s post hoc test which is based on the Analysis of Variance (ANOVA). Before the above parametric tests were used, a Shapiro-Wilk normality test^56^, F test, and Bartlett test^57^ were used to determine the assumptions of the t test and Tukey’s post hoc test^58, 59^, and all data in all groups met the requirements. All the above analyses were performed using R programming (version 3.3.2).

## Electronic supplementary material

Supplementary Information
