# Supplementary material for: The naphthalene catabolic protein NahG plays a key role in hexavalent chromium reduction in Pseudomonas brassicacearum LZ-4
Source: Sci Rep. 2017 Aug 29;7:9670. doi: 10.1038/s41598-017-10469-w (PMC5575117; doi:10.1038/s41598-017-10469-w)
Supplement: Supplementary file 1 — Supplementary Information [file 41598_2017_10469_MOESM1_ESM.doc]

**The naphthalene catabolic protein NahG plays a key role in hexavalent chromium reduction in** ***Pseudomonas brassicacearum* LZ-4**

**Haiying Huang ^a, #^, Xuanyu Tao ^a, #^, Yiming Jiang ^a^,, Aman Khan ^a^, Qi Wu ^a^ , Xuan Yu ^a^, Dan Wu ^b^, Yong Chen ^a^, Zhenmin Ling ^a^, Pu Liu ^a^, Xiangkai Li ^a^,***

Supplementary Figure legend and tables

Supplementary Figure S1 (A) Phylogenetic tree based on 16S rRNA gene sequences showing the relationships among various *Pseudomonas* species and the new isolate, strain LZ-4. Venn diagram (B) and collinearity analysis (C) comparing the genome of *Pseudomonas brassicacearum LZ-4* to other *Pseudomonas* genomes.

Supplementary Figure S2 (A) GO categories for all (LZ-all) unigenes. The figure was produced with WEGO. The results are summarized in three categories: biological process, cellular component, and molecular function. (B) COG function classification.

Supplementary Figure S3 Conversion of salicylate to catechol by NahG measured by HPLC. a: catechol standard solution, b: salicylate standard solution, c: FAD, d: NADH, e: salicylate+NADH+FAD+HEPES, pH 7.0 (control), f: NahG+salicylate+NADH+FAD+HEPES, pH 7.0 (experimental).

Supplementary Table.S1 Bacterial strains and plasmids used in this study

Supplementary Table.S2 General genome features of *Pseudomonas brassicacearum* LZ-4 supplementary

Supplementary Table S3. Result of Shapiro-Wilk normality test for the samples in Figure 3

Supplementary Table S4. Result of F test for the variance between two samples

Supplementary Table S5. Result of Shapiro-Wilk normality test for the samples in Figure 5

Supplementary Table S6. Result of Bartlett test of homogeneity of variance for the samples in Figure 5


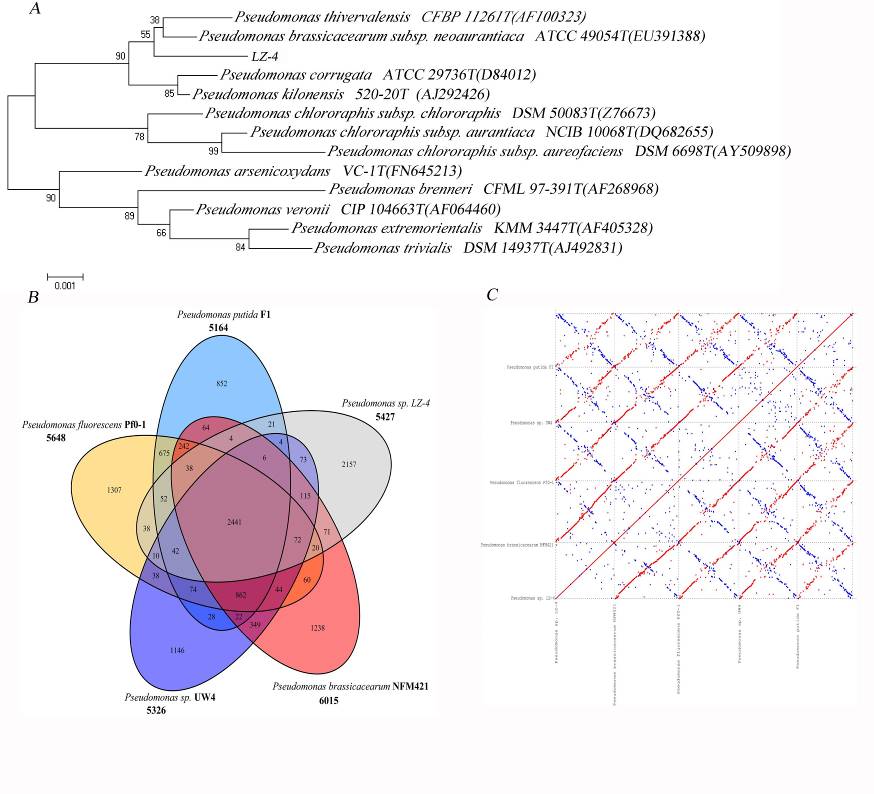


Supplementary Figure S1 (A) Phylogenetic tree based on 16S rRNA gene sequences showing the relationships among various *Pseudomonas* species and the new isolate, strain LZ-4. Venn diagram (B) and collinearity analysis (C) comparing the genome of *Pseudomonas brassicacearum LZ-4* to other *Pseudomonas* genomes. supplementary


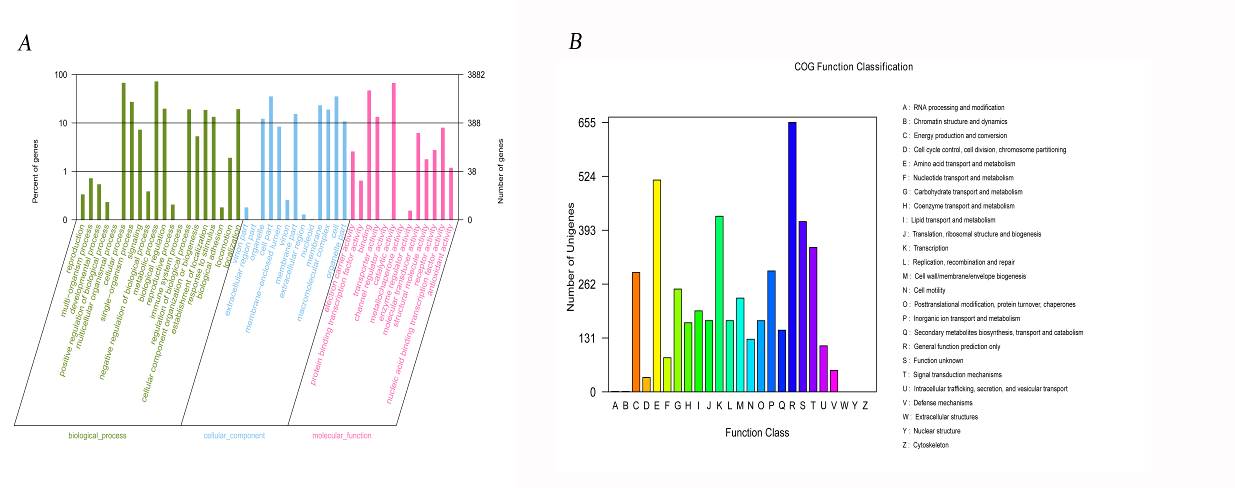


Supplementary Figure S2 (A) GO categories for all (LZ-all) unigenes. The figure was produced with WEGO. The results are summarized in three categories: biological process, cellular component, and molecular function. (B) COG function classification.


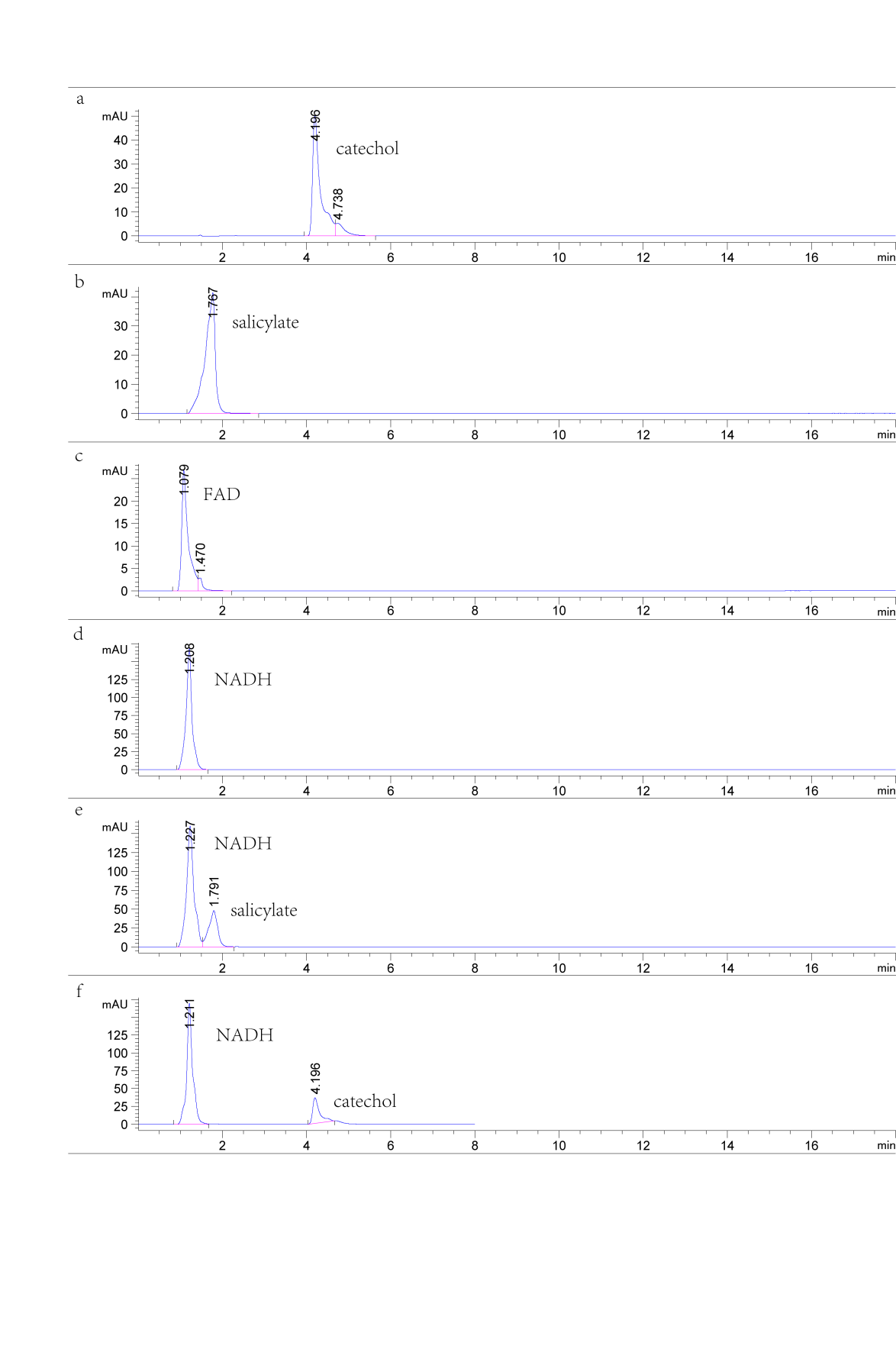


Supplementary Figure S3 Conversion of salicylate to catechol by NahG measured by HPLC. a: catechol standard solution, b: salicylate standard solution, c: FAD, d: NADH, e: salicylate+NADH+FAD+HEPES, pH 7.0 (control), f: NahG+salicylate+NADH+FAD+HEPES, pH 7.0 (experimental).

Supplementary Table S1 Bacterial strains and plasmids used in this study

| Strain or plasmid | Relevant characteristic | Source or reference |
| --- | --- | --- |
| Strains  *E. coli*  *DH5α* |  |  |
| S17-1 | *thi* pro res^-^ mod^+^ > Sm^r^ Tp^r^ rec*A1*RP-4-2[Tc::Mu;Km::Tn7] | Lab collection |
| *P. brassicacearum LZ-4* | Wild type of *P. brassicacearum* identiﬁed in contaminated soil | This study |
| Plasmids |  |  |
| pK18 *mobsacB* | Km^r^ , sacB lacZα mob | Lab collection |

Supplementary Table S2 General genome features of *Pseudomonas brassicacearum* LZ-4 supplementary

| **Feature** | **Number** |
| --- | --- |
| Size (bp) | 6,219,082 |
| G+C content (mol%) |  |
| Genome | 60.08 |
| Gene | 60.9 |
| Scaffolds | 98 |
| N50 (bp) | 177,781 |
| N90 (bp) | 50,044 |
| Contigs | 126 |
| N50 (bp) | 153,775 |
| N90 (bp) | 46,753 |
| Predicted ORFs | 5,464 |
| Genes |  |
| Total length | 6,219,082 |
| Average length | 979 |
| GO annotation | 3,882 |
| Protein-coding genes in COG | 4,638 |

All statistical methods for Figure 3 and Figure 5 were parametric tests.

In Figure 3, the Student’s t-test was used to determine significant differences between the control group and each treatment group. Before the t-test was performed, shapiro.test (Shapiro-Wilk normality test) and var. test (F test) from the “Stats” package in R programming were used to determine the normality and variances (Test for the t test assumptions), respectively. According to our preliminary test results (Tables S3 & S4), all samples were normally distributed and variances were equal between the control group (Cell-free group) and each treatment group (NADH, N, N+NADH, G, and G+NADH).

Supplementary Table S3. Result of Shapiro-Wilk normality test for the samples in Figure 3

| Group Name | Result of shapiro.test |
| --- | --- |
| Cell-free | W = 0.95434, p-value = 0.7433 |
| NADH | W = 0.92382, p-value = 0.5585 |
| N | W = 0.96198, p-value = 0.7914 |
| N+NADH | W = 0.95936, p-value = 0.7749 |
| G | W = 0.96027, p-value = 0.7806 |
| G+NADH | W = 0.90737, p-value = 0.4686 |

Supplementary Table S4. Result of F test for the variance between two samples

| Group Name | Result of var. test |
| --- | --- |
| Cell-free vs. NADH | p-value = 0.05016 |
| Cell-free vs. N | p-value = 0.5567 |
| Cell-free vs. N+NADH | p-value = 0.08242 |
| Cell-free vs. G | p-value = 0.4291 |
| Cell-free vs. G+NADH | p-value = 0.2295 |

For Figure 5, the Tukey’s post hoc test was used to determine significant differences among different groups. As mentioned, the Tukey’s post hoc test is based on the Analysis of Variance. Therefore, the Shapiro-Wilk normality test and Bartlett's test from the “Stats” package in R programming were used to test the normality and homogeneity of variances, respectively. According to preliminary results, all samples were normally distributed and variances were equal (Table S5 & S6).

Supplementary Table S5. Result of Shapiro-Wilk normality test for the samples in Figure 5

| Group Name | Result of shapiro.test |
| --- | --- |
| salicylate T | W = 0.79921, p-value = 0.1008 |
| salicylate C | W = 0.98084, p-value = 0.9069 |
| w/o salicylate T | W = 0.88491, p-value = 0.36 |
| w/o salicylate C | W = 0.98311, p-value = 0.92 |
| Catechol T | W = 0.93927, p-value = 0.6499 |
| Catechol C | W = 0.91264, p-value = 0.4965 |

Supplementary Table S6. Result of Bartlett test of homogeneity of variance for the samples in Figure 5

| Bartlett test of homogeneity of variances | Bartlett's K-squared = 6.4998, df = 5, p-value = 0.2606 |
| --- | --- |
